# Of inflammasomes and pathogens – sensing of microbes by the inflammasome

**DOI:** 10.1002/emmm.201201771

**Published:** 2013-05-13

**Authors:** Franz Bauernfeind, Veit Hornung

**Affiliations:** 1 Institute for Clinical Chemistry and Pharmacology, Unit for Clinical Biochemistry, University Hospital, University of BonnGermany; 2 Department of Internal Medicine III, University Hospital, University of BonnGermany

**Keywords:** caspase-1, inflammasome, NLRC4, NLRP3, pathogens

## Abstract

Inflammasomes are signalling platforms that sense a diverse range of microbial products and also a number of stress and damage associated endogenous signals. Inflammasome complexes can be formed by members of the Nod-like receptor family or the PYHIN family member AIM2. Upon formation, inflammasomes trigger proteolysis of caspase-1, which subsequently leads to a potent inflammatory response through the maturation and secretion of IL-1 family cytokines, which can be accompanied by an inflammatory cell death termed pyroptosis. Here, we review the sensing mechanisms of the currently characterized inflammasome complexes and discuss how they are involved in the innate immune response against microbial pathogens. We especially highlight recent advances in the molecular understanding of how microbial patterns are detected and discriminated from endogenous compounds by inflammasome sensors. Further, we review how inflammasomes contribute to the anti microbial host defense by cytokine-dependent and cell autonomous mechanisms.

## Introduction

**Figure 1:**
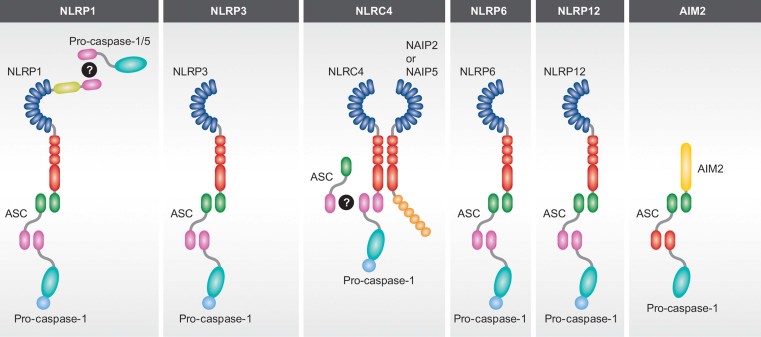
Assembled inflammasomes The nucleotide binding domain and LRR containing (NLR) family proteins NLRP1, NLRP3, NLRC4, NLRP6, NLRP12 and the pyhin protein AIM2 recruit and activate pro-caspase-1 indirectly through the bridging protein apoptosis-associated speck-like protein containing a CARD (ASC).

Physical barriers and immune defense systems have evolved to protect the host from microbial invasion. Acute inflammation is a response to infection or cellular disturbances by other means such as trauma. The initial inflammatory reaction limits harm to the body by directly impacting on microbial propagation and also by indicating the site of disturbed homeostasis to cells of the adaptive immune system. This immediate and innate immune effect is predominantly mediated via myeloid cells that sense conserved microbe associated molecular patterns (MAMPs) via a limited repertoire of germ-line encoded pattern recognition receptors (PRRs). MAMPs are mostly foreign structures and thus allow the specific sensing of invading organisms (Medzhitov, 2008). At the same time, some PRRs can also be triggered by endogenous substances that are formed or released during cell stress, perturbation of tissue homeostasis or metabolic imbalance. In analogy to the MAMP terminology, these signals are commonly referred to as damage associated molecular patterns (DAMPs). Activation of PRRs of the Toll-like receptor system (TLRs), RIG-I-like receptors (RLRs) or C-type lectin receptors (CLRs) initiates signalling cascades that result in pro-inflammatory gene expression. Additionally, PRR engagement sets off cascades that culminate in the proteolytical activation of inflammatory caspases. Hereby, a major inflammatory pathway is the cleavage and activation of caspase-1, which is initiated upon the formation of large multiprotein signalling platforms, the so-called inflammasomes. Activated caspase-1 proteolytically cleaves the cytokine precursors of interleukin-1β (IL-1β) and interleukin-18 (IL-18) to initiate a pro-inflammatory and antimicrobial response (Bauernfeind et al, 2011a). Various sensor proteins have been identified that can trigger the formation of inflammasome platforms. These inflammasome-forming PRRs, except for the DNA sensor AIM2, belong to the Nod-like receptor (NLR) family. NLRs are cytosolic PRRs with a tripartite domain architecture comprising of C-terminal leucine-rich repeats (LRRs) that are thought to sense microbial molecules or endogenous stress mediators; a central NACHT nucleoside triphosphatase domain that mediates NLR oligomerization and formation of the core structure of the inflammasome; and an N-terminal effector domain required for signal transduction. The latter can consist of a pyrin domain (PYD), a caspase recruitment domain (CARD) or a baculovirus inhibitor of apoptosis protein repeat (BIR) domain (Ting et al, 2008). Several NLRP proteins (NLR subfamily with N-terminal PYD domain) and the protein NLRC4 (NLR family CARD domain-containing protein 4) have been shown to form an inflammasome upon stimulation with the respective activator. Hereby, oligomerization of the inflammasome sensors NLRP1, NLRP3, NLRP6 or AIM2 (HIN200 protein family) allows interaction of the respective N-terminal PYD with the PYD of the protein ASC (Apoptosis associated speck-like containing a CARD domain) (Bauernfeind et al, 2011a; Elinav et al, 2011). ASC itself then recruits pro-caspase-1 via CARD-CARD interactions. According to its bipartite structure, composed of a PYD and CARD, ASC is often referred to as a bridging or adapter protein. The interaction of NLRPs, ASC and caspase-1 is based on homotypic interactions, which are common for death fold domains of the same subfamily, usually forming dimers or multimers. On the other hand, interactions across death domain subfamilies (*e.g.* CARD with PYD) are uncommon, even though somewhat surprising given their overall structural similarities. An overview of inflammasome proteins is depicted in Fig 1.

## Inflammasomes sense microbes in the cytosol to initiate an innate response

Phagocytic cells of the innate immune system, *e.g.* macrophages, respond to microbial infection using several mechanistically diverse signalling cascades. TLRs or CLRs, for example, sense extracellular or endolysosomal MAMPs and subsequently orchestrate immune responses by the transcriptional induction of anti-microbial effector molecules or mechanisms. At the same time, PRR activation can also activate effector mechanisms that function in the absence of *de novo* protein expression. For example, TLR4 ligation leads to rearrangement of the cytoskeleton and subsequently enhanced phagocytosis (Blander and Medzhitov, 2004). On the other hand, activated inflammasomes lead to proximity-induced autoproteolytic cleavage of the pro-enzyme caspase-1, resulting in its activation. Its thus generated subunits (p10 and p20) build tetramers to form the active cysteine protease, which converts the inactive IL-1β precursor to the C-terminal and active fragment. The pro-inflammatory cytokine IL-1β is an acute phase response mediator and promotes inflammation, vasodilation, hyperthermia and extravasation of immune cells, and is further involved in generation of Th17 cells (Dinarello, 2009). Notably, pro-IL-1β is expressed only at limiting amounts in resting cells and needs to be induced via the TLR signalling axis (Dinarello, 2009). The second well characterized caspase-1 substrate IL-18 is also expressed as a pro-cytokine but underlies only minimal transcriptional regulation. The caspase-1 cleaved C-terminal and secretable part of IL-18 promotes, together with IL-12, the production of IFN-γ in Th1, natural killer (NK) and cytotoxic T cells (Dinarello, 2009). Apart from its processive function, caspase-1 is required for an unconventional protein secretion pathway that is crucial for the release of IL-1β and various other target proteins (Keller et al, 2008). In addition, activity of caspase-1 in myeloid cells results in a special type of cell death, known as pyroptosis.

Pyroptotic cell death is programmed, caspase-1 dependent and pro-inflammatory. In contrast, apoptosis is not inflammatory, does not require caspase-1 yet is dependent on the effector caspases caspase-3, caspase-6 and caspase-7 (Bergsbaken et al, 2009; Kuida et al, 1995). Apoptotic cell death further results in regulated degradation and clearance of cellular contents (orchestrated disassembly of the cell). However, during pyroptosis cellular contents are released to the extracellular space and can induce inflammation (Bergsbaken et al, 2009). Thus, pyroptosis shares features of apoptosis and necrosis. In contrast to the former assumption that pyroptosis may be a pro-microbial mechanism employed by bacteria to destroy host phagocytes via induction of suicide in host cells, current data suggest, that pyroptosis rather functions as a host defense mechanism used to clear intracellular pathogens (Miao et al, 2010a).

## AIM2 inflammasome

The PYHIN family protein AIM2 is the only inflammasome sensor that does not belong to the NLR family, nevertheless some structural features are shared. AIM2 is characterized by the presence of an N-terminal PYD and a C-terminal HIN200 DNA-binding domain. Since AIM2 lacks a CARD, it essentially requires, similar to NLRPs, the bridging protein ASC to recruit caspase-1. Unlike other members of the PYHIN family, AIM2 is preferentially localized in the cytosol and operates as a direct intracellular sensor for cytosolic DNA (Fernandes-Alnemri et al, 2009; Hornung et al, 2009). So far, no substantial prerequisites for its ligand DNA have been described (*e.g.* sequence motifs or nucleotide modifications), beside the DNA needs to be double stranded and of more than 80 bp in length to accomplish sufficient AIM2 inflammasome formation to allow caspase-1 cleavage (Jin et al, 2012). Since AIM2 does not contain a NACHT domain, which could facilitate its multimerization, it was already initially speculated that the dsDNA sensed by AIM2 could provide the matrix for oligomerization (Bauernfeind et al, 2011a). This hypothesis is now tightened by the recently published crystal structure of the HIN domain of AIM2 in complex with its dsDNA ligand. Here, it was shown that non-sequence-specific DNA recognition is accomplished through electrostatic attraction between the positively charged HIN domain residues and the negatively charged dsDNA sugar-phosphate backbone. Upon binding, the pyrin domain is liberated from an intramolecular autoinhibitory complex (PYD/HIN domain complex). This facilitates the assembly of inflammasomes along the DNA staircase, whereby a HIN domain spans a spacing of 7–8 bp on each side of the dsDNA (Jin et al, 2012). As such, it is not surprising that transfected double stranded DNA (dsDNA) of either viral, procaryotic or mammalian origin was shown to bind to and activate AIM2 (Hornung et al, 2009). The fact that AIM2 activation does not require a certain sequence motif also implies that unintentional AIM2 activation by endogenous DNA is only prevented by AIM2's cytosolic compartmentalization, where dsDNA is not present under physiological conditions.

**Glossary**

**Apoptosis-associated speck-like protein containing a CARD (ASC)**

Adapter protein with a bipartite domain structure (PYD and CARD); ASC functions to recruit caspase-1 to oligomerizing NLRPs via homotypic domain interactions.

**Bacterial type III secretion (T3S) system**

A needle-like structure found in several gram-negative bacteria that is used to transfer bacterial molecules to eucaryotic cells.

**Caspases**

Cysteine proteases that play an essential role in apoptosis, necrosis, pyroptosis and inflammation.

**Danger-associated molecular patterns (DAMPs)**

Host-endogenous structures (*e.g.* modified proteins or nucleic acids) that can initiate an innate immune response under sterile conditions such as tissue damage.

**Inflammasomes**

Cytosolic, high molecular weight signaling platforms that function to activate inflammatory caspases.

**Microbe-associated molecular patterns (MAMPs)**

Microbial molecules recognized by pattern recognition receptors of the innate immune system.

**Pattern recognition receptors (PRRs)**

Germ-line encoded receptors of the innate immune system that recognize MAMPs or DAMPs.

**Phagosome**

Intracellular vesicle formed around a particle or microbe engulfed by phagocytosis. During their maturation, phagosomes fuse with lysosomes.

**Pyroptosis**

Special type of programmed and inflammatory cell death induced by caspase-1.

Although it was demonstrated in the past that endogenous DNA could accumulate or access cytosolic compartments when improperly degraded or insufficiently cleared from the extracellular space (Kawane et al, 2006), AIM2 activation through endogenous DNA has yet not been demonstrated in a physiological disease model. However, several microbial invaders can gain access to the cytosol of phagocytic cells and release foreign DNA to trigger AIM2 and caspase-1 activation. This has been shown for the DNA viruses vaccinia virus (VACV) and murine cytomegalovirus (MCMV) (Rathinam et al, 2010) as well as upon infection with bacterial pathogens such as *Francisella tularensis* (Fernandes-Alnemri et al, 2010; Rathinam et al, 2010), *Listeria monocytogenes* (Kim et al, 2010) and certain *Legionella pneumophila* strains (Ge et al, 2012). It is widely accepted and experimentally proven that free cytosolic microbial DNA is required to activate AIM2. However, it is currently unknown for *F. tularensis* infection, if the DNA is released while the bacterium is still within the phagosome or when it has entered the cytosol. *Francisella* virulence is closely linked to its ability to replicate in the host cytosol. Hypercytotoxic mutants that are defective for membrane proteins that affect bacterial stability or susceptibility for lysis show a higher release of cytosolic DNA, resulting in enhanced AIM2 activation and pyroptosis (Peng et al, 2011). *Legionella pneumophila*, the causative agent of Legionnaires' disease, resides in a distinct vacuole structure called Legionella-containing vacuole (LCV), which functions to avoid the fusion with the lysosome. Efficient *L. pneumophila* replication in the host macrophage requires a bacterial secretion system to translocate the effector SdhA to prevent host cell death. SdhA is most likely involved in membrane trafficking and was recently shown to maintain the integrity of the *LCV.* Strains lacking SdhA are defective for intracellular replication due to host cell death by DNA initiated pyroptosis (Creasey & Isberg, 2012; Ge et al, 2012). *L. monocytogenes*, on the other hand, replicates in the cytosol after escaping the phagosome by employing the pore forming cytolysin listeriolysin O (LLO) (Dramsi & Cossart, 2002) and loss of cell wall integrity through cytosolic bacteriolysis results in the release of bacterial DNA into the macrophage cytosol (Sauer et al, 2010; Warren et al, 2010). Thus, it seems that AIM2 gets activated whenever foreign DNA is present in the cytosol of inflammasome competent cells. As a consequence, bacterial pathogens seem to avoid AIM2 recognition through maintenance of bacterial structural integrity and protection from cytosolic host factors in vacuolar niches.

AIM2 deficiency increases the virulence of MCMV (Rathinam et al, 2010) and *F. tularensis* (Fernandes-Alnemri et al, 2010) *in vivo*. In a mouse model of MCMV infection, the AIM2 inflammasome mediates NK cell-dependent production of IFN-γ via IL-18 processing. These events were critical for the very early innate response to infection and are reflected by higher titers of MCMV in spleens of AIM2 deficient mice compared to littermate controls, however only for an early time point (Rathinam et al, 2010). Furthermore, AIM2 deficient mice are extremely susceptible to *F. tularensis* infections and display a higher bacterial burden in tissues, which is associated with greater mortality (Fernandes-Alnemri et al, 2010).

## The NLRC4 inflammasome

NLRC4 (formerly known as IPAF, Card12) has been shown to form an inflammasome upon infection of macrophages with various gram-negative bacteria such as *Salmonella typhimurium* (Mariathasan et al, 2004), *Legionella pneumophila* (Zamboni et al, 2006), *Shigella flexneri* (Suzuki et al, 2007) and *Pseudomonas aeruginosa* (Miao et al, 2008). NLRC4 contains an N-terminal CARD for signal transduction, a central NACHT domain, and a C-terminal LRR domain and it was shown to associate directly and specifically with the CARD domain of pro-caspase-1 through CARD–CARD interactions in overexpression systems (Poyet et al, 2001). Nevertheless, ASC seems to be required for cytokine processing by caspase-1, whereas cell death initiated by NLRC4 signalling occurs independent of ASC (Aachoui et al, 2013; Broz et al, 2010).

Early studies of NLRC4 inflammasome activation by gram-negative bacteria revealed that functional bacterial type III or type IV secretion systems seemed to be required for caspase-1 cleavage. Further experiments uncovered that *S. typhimurium* and *L. pneumophila* strains deficient for the protein flagellin, the main component of flagellum, were defective in their ability to activate NLRC4 (Miao et al, 2006; Zamboni et al, 2006). Flagellin independent NLRC4 activation by *P. aeruginosa* (Sutterwala et al, 2007) was later attributed to the sensing of compounds of the bacterial type III secretion (T3S) system (Miao et al, 2010b).

T3S systems function to deliver multiple bacterial effectors that confer virulence into the host cell to disrupt certain host processes to allow bacterial replication, particularly by circumventing an innate immune response (Gong & Shao, 2012). The T3S system apparatus consists of roughly 20 proteins building a bacterial membrane spanning multi-ring core base with an inner rod and a protruding extracellular needle-like structure that can insert into host cell membranes and form a conduit for bacterial effector proteins. Noteworthy, flagellin also enters the host cell by a translocation-associated T3S system (Buttner, 2012). Therefore, flagellin-mediated stimulation of the inflammasome pathway by bacterial mutants harbouring genetic mutations that disrupt T3S systems is usually absent or significantly reduced (Miao et al, 2006).

## Mechanism of NLRC4/NAIP activation

Together with the observation that even purified and transfected flagellin has the ability to activate NLRC4 (Franchi et al, 2006; Miao et al, 2006), these early studies suggested a model of direct sensing of the MAMP flagellin through NLRC4 after cytosolic delivery via T3S systems. However, several points challenged this assumption. (1) Direct interaction of NLRC4 and flagellin could not be shown (Franchi et al, 2006). (2) Infection with *Shigella flexneri*, an aflagellated bacterium, also induced NLRC4 inflammasomes whereby the presence of its T3S system was still required (Suzuki et al, 2007). (3) Wild type C57BL/6 macrophages restricted *L. pneumophila* replication, whereas A/J strains were highly permissive. The genomic locus accounting for this phenotype was mapped to a region containing NAIP proteins (NLR family, apoptosis inhibitory protein) (Kofoed & Vance, 2011) and NAIP5 was shown not only to restrict *L. pneumophila* replication but also to be required for caspase-1 activation and pyroptosis upon *L. pneumophila* infection (Lightfield et al, 2008). (4) The C-terminal portion spanning 35 amino acids of flagellin, forming an alpha helical domain was shown to rely on NAIP5, whereas full length flagellin did not require NAIP5 when massively overexpressed (Lightfield et al, 2008). Additionally, the *P. aeruginosa* mutant PAK Δ*fliC*, which is deficient in flagellin, is still capable of activating caspase-1 in an NLRC4-dependent manner without a requirement for NAIP5 (Sutterwala et al, 2007). (5) It could be shown that besides flagellin, a second bacterial protein that is present in many bacterial pathogens, the inner rod protein PrgJ of T3S systems is sensed by NLRC4 without the requirement for NAIP5 (Miao et al, 2010b).

**Figure 2:**
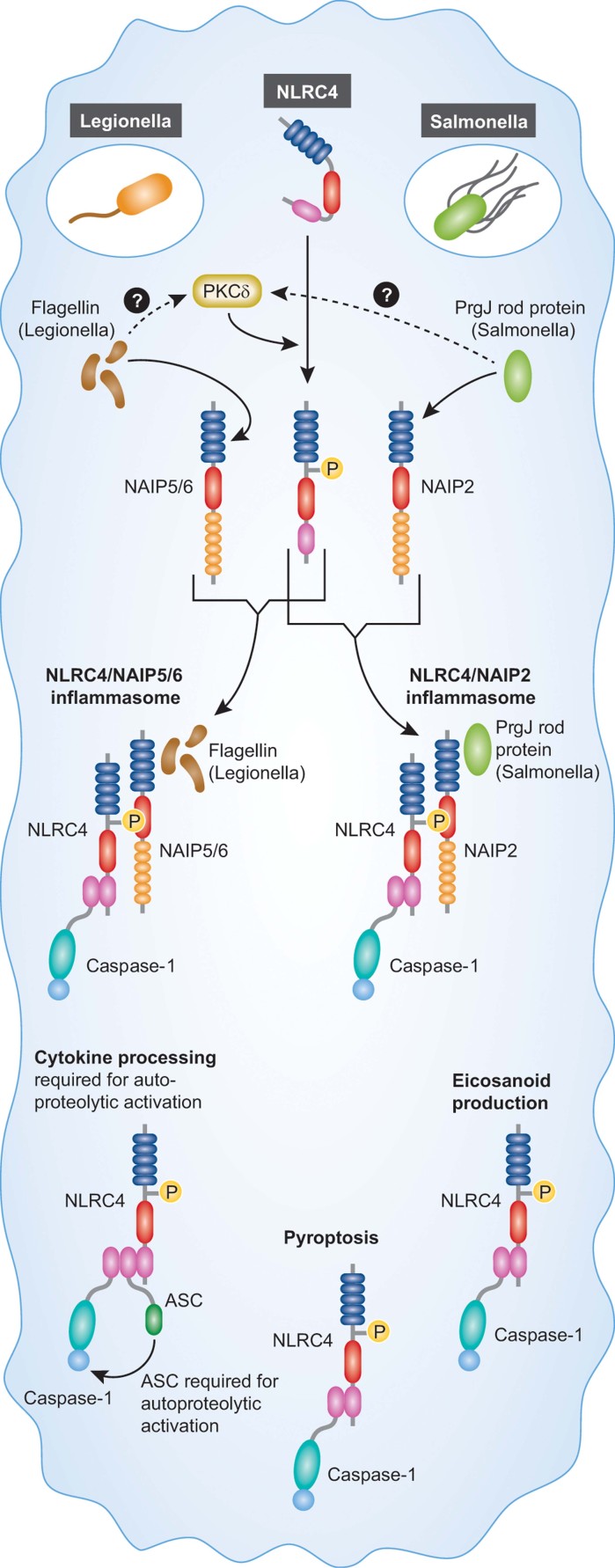
The NLRC4/NAIP inflammasome NAIP proteins sense flagellin or structurally related subunits of T3S systems in the cytosol of host macrophages. Several flagellin species directly bind to the C-terminal part of NAIP5 or NAIP6, whereas PrgJ binds to NAIP2. Ligand binding of NAIPs results in oligomerization with PKCδ-phosphorylated NLRC4 to recruit caspase-1. The presence of caspase-1 is required for various inflammasome effector mechanisms, however catalytic activity is only needed for processing of IL-1β and IL-18. Phosphorylation of NLRC4 by PKCδ requires the cytosolic presence of flagellin or T3SS recognition, yet the exact mechanistics of this process are currently unclear.

A sequence of recent studies has now clarified several of these issues, at least for the murine system (Kofoed & Vance, 2011; Zhao et al, 2011). Reconstitution of inflammasomes by overexpression of NLRC4, NAIPs and bacterial compounds in HEK cells followed by biochemical analysis led to a conclusive NLRC4 inflammasome model (Fig 2). Here, NLRC4 does not function as a receptor but distinct NAIP proteins bind to the MAMPs flagellin or PrgJ and associate with NLRC4. NAIP5 directly binds the C-terminal part of *Salmonella* flagellin and NAIP2 interacts with PrgJ-like proteins (PrgJ of *S. typhimurium* and BsaK of *B. thailandensis*) (Kofoed & Vance, 2011; Zhao et al, 2011). NAIP6 can also bind flagellin but its physiological role is unclear due to its expression at limiting amounts (Zhao et al, 2011). Ligand binding of NAIPs facilitates their interaction with NLRC4 and allows the latter to oligomerize and assemble an inflammasome. Reconstitution experiments with truncated dominant positive variants clearly demonstrated that NAIPs act upstream of NLRC4 and proposed that a ‘folding back’ of the LRR domain in the native state blocks the NBD domain responsible for oligomerization (Fig 2). Ligand binding could induce a conformational change of NAIPs and release the NBD from the autoinhibitory state (Gong & Shao, 2012; Kofoed & Vance, 2011) to allow assembly of an NLRC4/NAIP inflammasome, which also involves phosphorylation of the serine residue 553 of NLRC4 by kinases such as PKCδ (Qu et al, 2012).

Thus, distinct NAIP proteins seem to allow the NLRC4 inflammasome to differentiate between its bacterial ligands. Hereby, NAIPs that were previously believed to function as inhibitors of apoptosis, act as receptors.

C57BL/6 mice express four of the seven murine NAIP genes identified (NAIP1,2,5 and 6), whereas humans seem to be equipped with only one functional NAIP protein. Human NAIP can detect neither flagellin nor inner rod proteins of T3S systems. Instead, it recognizes the needle subunits (CprI or homologs) of bacterial T3S systems of *EHEC*, *B. thailandensis*, *P. aeruginosa*, *S. typhimurium* and *S. flexneri* (Zhao et al, 2011). In this regard, further studies are required to precisely elucidate differences between human and murine NLRC4 inflammasomes.

## NLRC4 inflammasome *in vivo*

Hosts may use detection of bacterial flagellin to discriminate commensal from pathogenic strains. For example, flagellin differs between bacterial species. While flagellin from *E. coli* fails to trigger NLRC4 activation, even if delivered to the host cytosol (Ren et al, 2006), *H. pylori* flagellin can activate the NLRC4 inflammasome. In line with this, the ability of flagellins from ten different bacterial pathogens to bind to NAIP5 correlates well with their inflammasome stimulating activity (Zhao et al, 2011). The bacterial T3S system delivering virulence factors is structurally related to the bacterial flagellin system. Not surprisingly, since T3S system delivered virulence factors can modulate host-signalling pathways for bacterial benefit, the mammalian system has developed strategies to detect T3S activity via NLRC4 (Miao et al, 2006, 2008).

The NLRC4 sensing machinery has been shown to contribute to pathogen defense by several distinct mechanisms. Conventional inflammasome activation resulting in IL-1β and IL-18 release was studied *in vivo* for diverse bacterial strains. For example, caspase-1 induced release of IL-1β and IL-18 is essential for *S. flexneri* defense in mice (Sansonetti et al, 2000), whereas it is not clear to what extent this depends on NLRC4. However, IL-1β and IL-18 are only partially required for the NAIP/NLRC4 dependent defense against *S. typhimurium*, *L. pneumophila* and *B. thailandensis in vivo* (Miao et al, 2010a). In this regard, a novel cell intrinsic, cytokine independent signalling output of NLRC4 activation was recently characterized. Von Moltke et al. identified eicosanoids as previously unrecognized inflammasome effectors. Activation of the NLRC4/caspase-1 axis through FlaTox (a synthetic fusion protein of *Legionella* flagellin and anthrax lethal factor delivered through anthrax protective antigen) specifically resulted in a rapid induction of inflammatory lipid mediators, a so called “eicosanoid storm”. Mice deficient in cyclooxygenase-1, the critical enzyme in prostaglandin biosynthesis, were resistant to eicosanoid caused inflammation and vascular fluid loss (von Moltke et al, 2012). The relevance of these results needs to be verified in more physiological models, especially since NLRC4 activation was achieved by a highly dosed and artificial ligand. Nevertheless, this study disclosed, that the realm of signalling outputs of inflammasomes might be much broader than previously thought.

On the cell autonomous level, NLRC4 induced activation can contribute to antimicrobial defense by degradation of pathogens inside macrophages, which is promoted by the fusion of the LCV, an endoplasmic reticulum (ER) derived compartment resembling an immature autophagosome where *L. pneumophila* replicates, with the lysosome. This effect is possibly mediated by caspase-7 acting downstream of caspase-1 (Akhter et al, 2009). The fusion of the pathogen-enclosing compartment with the lysosome is facilitated by the NLRC4/caspase-1 axis and thereby restricts *Legionella* replication (Amer et al, 2006).

Pyroptosis provides another clearing mechanism. *S. typhimurium* strains modified to persistently express flagellin induced caspase-1 dependent pyroptotic cell death. Bacteria released from pyroptotic macrophages were exposed to and taken up by neutrophils and killed by their reactive oxygen species (ROS). This has been shown to be a caspase-1 dependent process that occurs independently of IL-1β and IL-18 *in vivo* (Miao et al, 2010a).

## NLRP1 inflammasome

Although NLRP1 has been the first protein described to form an inflammasome with the minimal requirement of caspase-1, caspase-5 and ASC (Martinon et al, 2002), its mechanism of activation remains poorly understood. NLRP1 structurally differs from other NLRs in its additional C-terminal extension consisting of a domain with unknown function (function to find domain, FIIND) and a CARD domain. Human NLRP1 forms an ASC-dependent inflammasome, whereas mouse NLRP1 may activate caspase-1 in an ASC-independent manner (Hsu et al, 2008). The best-characterized elicitor of NLRP1 activation is anthrax lethal toxin (Boyden & Dietrich, 2006), the major virulence factor of *Bacillus anthracis*. Its protective antigen (PA) subunit allows the effector subunit lethal factor (LF) to enter the cell cytosol. LF activates caspase-1 and induces rapid cell death via NLRP1. Since inactive but structurally virtually identical mutants of LF fail to activate caspase-1, it is most likely, that LF does not directly bind to NLRP1 (Fink et al, 2008). Indeed, the endoprotease activity of LF is required to alert NLRP1 activation. LF has the capability of cleaving cytosolic substrates, a process that additionally requires Ca^2+^ flux and probably also proteasome activity (Fink et al, 2008). Thus, it was proposed that LF could be sensed by NLRP1 through the presence of cleaved host substrates rather than direct binding (Fink et al, 2008). Some evidence also suggests that NLRP1 undergoes autoproteolytic cleavage at a conserved motif within its FIIND (D'Osualdo et al, 2011) and that direct cleavage of a conserved motif in the FIIND by LF may present a necessary but not sufficient step for NLRP1 activation (Hellmich et al, 2012; Levinsohn et al, 2012). Nevertheless, the precise mechanism by which LT triggers the NLRP1 inflammasome remains unclear. Humans harbour only one NLRP1 gene, whereas three paralogues (Nlrp1a, b, c) are present in mice (Boyden & Dietrich, 2006). Moreover, the murine Nlrp1b gene is highly polymorphic and different mouse strain variants of Nlrp1b confer susceptibility to LF induced caspase-1 activation (Boyden & Dietrich, 2006). Nlrp1b activation by LT represents an essential host defense mechanism in the control of *B. anthracis* infection. Caspase-1 induced pyroptosis of infected macrophages permits self-elimination of affected cells and initiation of an antimicrobial neutrophilic reaction, which also involves IL-1β (Terra et al, 2010; Welkos et al, 1986, 1989).

Recently, a hyperactive mutation in the murine Nlrp1a paralogue was identified to trigger a systemic caspase-1 dependent inflammatory response *in vivo* (Masters et al, 2012). IL-1β was critical for the multiorgan neutrophilic disease, whereas ASC was dispensable for the inflammatory phenotype. Certainly, the exact Nlrp1a ligand or activation signal needs yet to be elucidated, but studies in Nlrp1a deficient mice suggest a role for Nlrp1a in triggering pyroptosis of haematopoietic progenitor cells during periods of haematopoietic stress induced by chemotherapy or infection (Masters et al, 2012). Whether this observation also has implications for human immunology and haematopoiesis needs to be investigated.

## NLRP3 inflammasome

**Figure 3:**
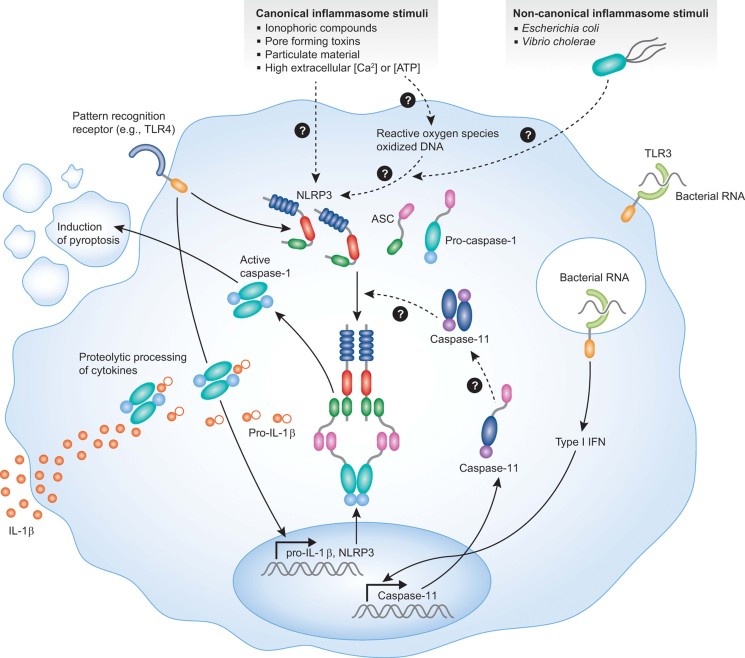
Mechanisms resulting in NLRP3 activation Transcription of IL-1β and NLRP3, which expression is critical, is induced by TLR signalling. At the same time, NLRP3 can be primed by a TLR-dependent,but transcription-independent signalling event. Various stimuli can activate NLRP3 that is believed to be present in an auto-inhibited state. Activated NLRP3 presumably forms multiprotein aggregates with the adaptor protein ASC and recruits caspase-1. Autocatalytically activated caspase-1 in turn processes pro-IL-1β to an active and secretable form. A plausible common downstream mechanism or signalling mediator that integrates the various processes induced by a plethora of canonical NLRP3 stimuli has not been identified but various models including as ROS production or Ca^2+^ release from the endoplasmatic reticulum were proposed. In contrast to these stimuli, non-canonical stimuli such as live gram-negative bacteria additionally require caspase-11 for full caspase-1 activation. The activity of caspase-11 is transcriptionally regulated by type I IFN signalling that is simultaneously engaged by bacterial compounds via TLR activation. The molecular mechanisms linking caspase-11 to caspase-1 have not been entirely identified.

NLRP3 (Nalp3, cryopyrin) forms an inflammasome with ASC and caspase-1 (Agostini et al, 2004) and has been the most extensively studied NLR member due to a wide array of activators of microbial and non-microbial origin (Fig 3). The NLRP3 inflammasome has been implicated in sensing a plethora of pathogenic bacteria including *S. aureus* and *E. coli* (Rathinam et al, 2012), viral pathogens such as *Influenza A virus* (Allen et al, 2009; Thomas et al, 2009) or *vesicular stomatitis virus* (Rajan et al, 2011) and the fungal pathogens *Candida albicans* (Gross et al, 2009) and *Aspergillus fumigatus* (Said-Sadier et al, 2010). Also parasites, such as *Schistosoma mansoni* (Ritter et al, 2010) or *Dermatophagoides pteronyssinus* (Dai et al, 2011) have been shown to activate NLRP3 (for a comprehensive list of NLRP3 activating pathogens please refer to Bauernfeind et al, 2011a; Franchi et al, 2012b; Lamkanfi & Dixit, 2009a). Further effort to identify NLRP3 activating compounds of pathogens revealed that several membrane pore forming or ionophoric agents of bacteria (*e.g.* LLO or Nigericin (Mariathasan et al, 2006), Streptolysin (Harder et al, 2009)) or viruses (M2 channel of *Influenza A* virus (Ichinohe et al, 2010)) activate NLRP3. Additionally, endogenous DAMPs such as extracellular ATP, gout associated uric acid crystals (Martinon et al, 2006), fibrillar amyloid beta (Halle et al, 2008) or cholesterol crystals (Duewell et al, 2010), and also environmental products like silica crystals (Hornung et al, 2008), asbestos fibers (Dostert et al, 2008) and nanomaterials (Yazdi et al, 2010) have been shown to give rise to NLRP3 inflammasome activation. The wide and structurally diverse array of agents inducing NLRP3 activation implies that a shared, but yet unknown mechanism upstream of NLRP3 enables its oligomerization. In line with this hypothesis, it was suggested that NLRP3 could monitor a host-derived DAMP that is produced or released as a consequence of cellular injury of microbial or non-microbial NLRP3 activators (Lamkanfi & Dixit, 2009b). However, there is yet no evidence for the binding of a specific ligand, serving as a common denominator, to the LRR domain of NLRP3. Despite unclear upstream mechanisms, NLRP3 is expressed at limiting levels in macrophages or dendritic cells, possibly to avoid unintended inflammation, and requires a PRR-dependent priming signal via TLRs as a first necessity for its subsequent activation (Bauernfeind et al, 2009; Juliana et al, 2012). A number of putative mechanisms have been postulated to account for the activation step, which includes phagolysosomal destabilization, generation of ROS or the induction of transmembrane ion fluxes.

## Putative models of NLRP3 activation

Macrophages activate a number of enzymes to degrade engulfed material in lysosomal compartments. However, unproductive phagocytosis of endogenous molecules such as uric acid crystals leads to lysosomal disintegration and release of lysosomal contents into the cytosol. This process requires cytoskeletal dynamics, acidification of endolysosomes and correlates with NLRP3 activation (Hornung et al, 2008). Hereby, the critical process for triggering NLRP3 seems to be the release of lysosomal contents, since specific rupture of lysosomal membranes by other means is sufficient to activate NLRP3 (Hornung et al, 2008). Moreover, based on inhibitor experiments it was suggested that release of cathepsin-B into the cytosol was an essential step in this process. However, cathepsin-B deficient macrophages are not completely defective in driving crystal induced NLRP3 activation (Dostert et al, 2009). It is possible, that redundant cathepsins or other leaking lysosomal proteases trigger NLRP3 via processing of yet-unidentified substrates. However, this upstream mechanism is specific for particles, as NLRP3 activation by bacterial toxins or ATP occurs independent of phagocytosis (Hornung et al, 2008). At the same time, it is not clear how lysosomal disintegration is linked to the proximal steps of NLRP3 activation.

Another model is based on the observation that several NLRP3 activators such as crystals, ATP and Nigericin induce mitochondria-derived ROS production in macrophages. ROS can cause oxidative modifications to nucleic acids, lipids and proteins. As such, induction of ROS production by NLRP3 stimuli was implicated in NLRP3 activation (Cruz et al, 2007; Zhou et al, 2011), as well as loss of membrane potential and release of oxidized mitochondrial DNA into the cytosol (Shimada et al, 2012). However, TLR signalling induced ROS production fails to trigger NLRP3. Concurrently, ROS production was also implicated in the TLR dependent priming step, rather than in the second, NLRP3 activating signal (Bauernfeind et al, 2011b).

It has also been described that potassium efflux is required for NLRP3 activation by various stimuli, a notion that was mainly attributed to the fact that potassium ionophores (*e.g.* Nigericin) are strong activators of the NLRP3 inflammasome, whereas high extracellular potassium concentrations that block K^+^ efflux potently inhibit NLRP3 activation (Petrilli et al, 2007). However, even though NLRP3 appears to be most sensitive to K^+^ efflux inhibition, blocking of caspase-1 cleavage by raising concentrations of extracellular potassium is not specific for the NLRP3 inflammasome (Fink et al, 2008). Furthermore, ion fluxes through the purinergic P2X7 receptor associated pore and pores formed by bacterial toxins are not specific for potassium, as cellular concentrations of H^+^, Na^+^ and Ca^2+^ ions are also modulated.

Indeed, calcium signalling was recently suggested to be critical in the NLRP3 activation process. Several NLRP3 activators release Ca^2+^ from the ER (Murakami et al, 2012) and Ca^2+^ release was suggested to be a common proximal step in activation of NLRP3. However, Ca^2+^ signalling alone cannot be sufficient for NLRP3 activation since the Ca^2+^ ionophore ionomycin fails to activate NLRP3 (Murakami et al, 2012). Another study implicated the membrane-associated calcium sensing receptor (CaSR) in NLRP3 activation (Lee et al, 2012). Here, it was proposed that ion perturbation leads to recognition of elevated extracellular Ca^2+^ via CaSR. CaSR signalling then results in the activation of phospholipase C, which induces intracellular Ca^2+^ release from the ER. On the other hand CaSR signalling inhibits adenylate cyclase and subsequently reduces cAMP levels. The combination of increased cytosolic Ca^2+^ and decreased cAMP was suggested to activate NLRP3 (Lee et al, 2012).

At the protein level, deubiquitination of NLRP3 is a recently described potential molecular mechanism involved in its activation process (Juliana et al, 2012; Py et al, 2013), whereas thiol nitrosylation of NLRP3 through nitric oxide inducing agents such as pharmacological substances or lymphocyte derived IFN-γ down-modulates NLRP3 activity (Hernandez-Cuellar et al, 2012; Mishra et al, 2013).

Despite the above-mentioned mechanisms that were reported to trigger NLRP3 inflammasome formation, especially the proximal steps in NLRP3 activation are not well understood. Altogether, NLRP3 seems to be an integrator of cellular stress that can result from a large number of different agents. Whether, these signals converge on a common signalling cascade or a DAMP molecule proximal to NLRP3 or whether they activate NLRP3 through independent routes needs to be determined.

## NLRP3 inflammasome *in vivo*

Regardless of the exact mechanism governing the activation of the NLRP3 inflammasome, it was recently described, that caspase-1 cleavage via NLRP3 activation following *E. coli*, *C. rodentium* and *V. cholerae* infection additionally requires murine caspase-11 (Kayagaki et al, 2011). Moreover, it seems that all live gram-negative bacteria engage this pathway (Rathinam et al, 2012). To distinguish it from the “canonical” NLRP3 activation pathway that is engaged upon ATP or Nigericin stimulation, this inflammasome axis requiring caspase-11 was termed “non-canonical”. Caspase-11 deficient mice are markedly resistant to lethal doses of LPS, whereas caspase-1 deficiency is only partially protective (Kayagaki et al, 2011), thus caspase-11 may promote tissue damage and lethality independently of caspase-1. Additionally, caspase-11 protects animals from lethal infection by *B. thailandensis* and *B. pseudomallei*, two bacterial strains that have the ability to invade the cytosol (Aachoui et al, 2013). The exact function of caspase-11 needs yet to be elucidated, but it seems that it synergizes with the assembled NLRP3 inflammasome to regulate caspase-1 activation and induces caspase-1 independent cell death. Caspase-11 itself is regulated at the transcriptional level via type I IFN signalling induced by the TLR4/TRIF axis, but also MyD88 deficient macrophages show a delay in pro-caspase-11 induction (Broz et al, 2012). It was described that induction of caspase-11 expression may be sufficient for its cleavage through auto-activation (Rathinam et al, 2012), yet this is in conflict with the recent finding that LPS or IFN-β treatment without infection (Broz et al, 2012) or priming with avirulent bacteria (Case et al, 2013) does not result in caspase-11 activation in primary cells. This raises the possibility that additional microbial signals and yet to be identified host receptor platforms might be required for the activation of caspase-11. The *in vivo* relevance of the NLRP3 inflammasome is well documented by its role in hereditary autoinflammatory syndromes (Masters et al, 2009) and by its role in the progression of metabolic diseases that are accompanied with inflammation such as atherosclerosis, diabetes and gout (Wen et al, 2012). Studying the role of inflammasomes in murine infection models revealed critical roles for ASC and caspase-1 in the host defense against *S. aureus* and *V. cholerae*, whereas NLRP3 was surprisingly dispensable, even though these pathogens selectively trigger NLRP3 *in vitro*. The same applies for several other bacterial strains. This discrepancy might be due to redundancy *in vivo*, where a pathogen may employ multiple inflammasomes that contribute to host defense. Additionally, the contribution of the potential sensors AIM2, NLRC4 and NLRP3 may depend on the experimental model employed.

Nevertheless, it seems that NLRP3 can selectively elicit a protective antibacterial response as exemplified in a *S. pneumoniae* lung infection model, where NLRP3 deficient mice exhibited higher mortality and increased bacterial loads (Witzenrath et al, 2011). On the other hand, NLRP3 mediated inflammation in pneumococcal meningitis also conferred damage to the host tissue and NLRP3 deficient mice show an improved clinical outcome due to decreased brain inflammation (Hoegen et al, 2011).

The contribution of NLRP3 to *Influenza A* antiviral immunity is controversial. *Influenza A* virus has been shown to activate NLRP3 through the proton-selective ion channel M2 (Ichinohe et al, 2010) resulting in a NLRP3 dependent inflammatory response *in vivo* (Ichinohe et al, 2009). However, results regarding the influence of NLRP3 on viral burden and survival of viral infection are conflictive (Allen et al, 2009; Ichinohe et al, 2009; Thomas et al, 2009). In contrast, NLRP3 is critically involved in antifungal immunity against *Candida albicans* infection, having a clear protective effect in this context (Gross et al, 2009; Joly et al, 2009).

## NLRP6 inflammasome

NLRP6 (also called Pypaf5) associates with ASC and caspase-1 and can additionally trigger the activation of NFκB in transient overexpression systems (Grenier et al, 2002). There is currently no evidence of NLRP6 being activated by a specific pathogen-derived compound. However, NLRP6 activation seems to maintain the colonic microbiota via the ASC/caspase-1/IL-18 axis (Elinav et al, 2011). Mice lacking NLRP6 exhibit quantitative and qualitative changes in the gut microbial ecology with an outgrowth of *Prevotellaceae* and TM7, and reductions in *Lactobacilli* and *Firmicutes*. Similar imbalances were seen in ASC, caspase-1 and IL-18 deficient mice. Altered communities in the gut triggered susceptibility to colitis and spontaneous inflammation in the intestine by the recruitment of inflammatory cells via CCL5 (Elinav et al, 2011). Interestingly, NLRP6 inflammasome function in the gut is confined to epithelial cells. While ASC and caspase-1 are highly expressed in both haematopoietic and epithelial cells, NLRP6 expression is limited to the epithelial compartment (Elinav et al, 2011). The signal triggering the NLRP6 inflammasome in epithelial cells is currently unknown. However, it appears unlikely that NLRP6 can differentiate between innumerable bacterial, archaeal and eucaryotic phylotypes, especially since some act as commensals and only a few as pathogens. Instead, it was speculated that yet unidentified molecules signalling tissue disintegration might drive NLRP6 assembly.

It has also been shown that modulation of the gut flora by NLRP6 (in addition to NLRP3) negatively regulates the progression of non-alcoholic fatty liver disease and other aspects of the metabolic syndrome (Henao-Mejia et al, 2012). But it needs to be determined whether enhanced tumourigenesis and impaired self-renewal of epithelial cells in the intestine of NLRP6 deficient mice (Normand et al, 2011) is also a consequence of reduced inflammasome activation and the harmful imbalanced microbiota in the gut.

In sharp contrast to its protective effect in the gastrointestinal tract, NLRP6 has a detrimental role during systemic infections with gram-positive and gram-negative microbes. This is illustrated by a better survival and bacterial clearance of NLRP6 deficient mice after *L. monocytogenes*, *S. typhimurium* or *E. coli* infections (Anand et al, 2012). The presence of NLRP6 inhibited the influx of monocytes and neutrophils to the circulation and the peritoneum due to dampened cytokine and chemokine production during bacterial infections by negative regulation of TLR-induced canonical NF-κB and MAPK activation (Anand et al, 2012). Thus, NLRP6 was shown to exert, in contrast to its initial description (Grenier et al, 2002), an inhibitory function in the NF-κB pathway. Altogether, NLRP6 seems to have a protective role via the TLR axis under conditions where strong inflammatory responses are detrimental, whereas its inflammasome function is essential for maintaining intestinal homeostasis.

## NLRP12 inflammasome

NLRP12 forms an inflammasome with ASC and caspase-1 to mature IL-1β (Wang et al, 2002). Mutations of the NLRP12 coding sequence in the human genome are associated with an IL-1 mediated inflammatory disease (Jeru et al, 2008). Our knowledge of the role of NLRP12 in health and disease is limited, yet recent data suggest that NLRP12 is important for the recognition of *Yersinia pestis*, the causative agent of plague. Hereby, NLRP12 controls caspase-1 cleavage, IL-1β and IL-18 secretion upon *Y. pestis* infection of macrophages (Vladimer et al, 2012). The precise nature of the NLRP12 ligand is currently unknown, but since NLRP12 activation seems to require a functional T3S system (Vladimer et al, 2012), a bacterial virulence factor getting access to the host cytosol may be required to directly activate NLRP12 or alter host signalling pathways. Regardless the exact activation mechanism, NLRP12 driven IL-18 secretion and associated IFN-γ production essentially contribute to resistance against *Y. pestis* infection in mice. NLRP12 deficient mice showed higher mortality and bacterial loads after infection (Vladimer et al, 2012). In addition to forming an inflammasome, NLRP12 was implicated in suppression of intestinal inflammation and tumourigenesis through negative regulation of NF-κB signalling (Allen et al, 2012).

## Conclusions and future perspectives

Since the discovery of caspase-1 as the IL-1β converting enzyme, remarkable progress has been made in the identification and characterization of inflammasome sensors and their effector mechanisms. Even though the molecular events resulting in inflammasome activation have become clearer for certain sensors (*e.g.* AIM2 or NLRC4), the precise mechanisms of activation remain largely unknown for the majority of inflammasome complexes. Due to its prominent role in the innate immune response against microbial pathogens and its role in metabolic diseases and autoinflammatory disorders, elucidating the mechanisms of NLRP3 activation will be of great interest. However, the fact that NLRP3 signalling is tightly connected with the TLR pathway and the notion that NLRP3 appears to be indirectly activated by a yet to be identified endogenous stress signal complicates this venture.

With the growing number of inflammasome forming NLRs and their activators, regulatory mechanisms will also become a central focus of attention. For example, viruses have developed strategies to evade inflammasome recognition through expression of caspase-1 protease inhibitors (Ray et al, 1992; Young et al, 2000) or proteins homologous to host pyrin only proteins (POPs), that bind to ASC and inhibit inflammasome formation (Johnston et al, 2005). Bacterial microbes rather seem to use evasion strategies interfering with the expression of MAMPs, such as flagellin or T3S systems that are markedly down regulated during systemic infections (Broz & Monack, 2011). However, it is also possible that inflammasome-specific antagonistic mechanisms exist here as well.

**Pending issues**

Elucidation of differences between murine and human inflammasomes.

Clarification of the precise molecular mechanism of NLRP3 activation.

Better understanding of the physiologic role of the less well known NLRP6 and NLRP12 inflammasomes and identification of their respective ligands.

Clarification of the activation process of caspase-11.

Precise role of uncharacterized NAIPs and their role in sensing microbial pathogens.

Identification of evasion strategies developed by pathogens to circumvent inflammasome recognition.

Systematic exploration of inflammasome functionality in non-myeloid cells.

At the cellular level, the role of inflammasomes in pathogen sensing have mainly been studied in bone-marrow derived myeloid cells. Recent evidence, however, suggests that the local microenvironment plays a profound role in the sensitivity of tissue-resident macrophages towards inflammasome activating pathogens (Franchi et al, 2012a). As such, it will be interesting to revisit some earlier studies on the interaction of microbial pathogens and myeloid cells within their physiological local context. Moreover, knowing other cytokine-independent inflammasome effector mechanisms, such as pyroptosis or eicosanoid-mediated effects, it will be interesting to dissect the functional relevance of these different effector mechanisms in anti-microbial defense.
